# Fracture resistance of posterior teeth restored with modern restorative materials

**DOI:** 10.1016/S1674-8301(11)60055-9

**Published:** 2011-11

**Authors:** Ibrahim M. Hamouda, Salah H. Shehata

**Affiliations:** a Department of Biomaterials;; b Department of Conservative Dentistry, Mansoura University, Al-Gomhoria St, Mansoura 35516, Egypt.

**Keywords:** fracture resistance, composite resin, adhesives, mesio-occlusal-distal (MOD) cavities

## Abstract

We studied the fracture resistance of maxillary premolars restored with recent restorative materials. Fifty maxillary premolars were divided into five groups: Group 1 were unprepared teeth; Group 2 were teeth prepared without restoration; Group 3 were teeth restored with tetric ceram HB; Group 4 were teeth restored with InTen S; and Group 5 were teeth restored with Admira. The samples were tested using a universal testing machine. Peak loads at fracture were recorded. The teeth restored with Admira had the highest fracture resistance followed by those restored with InTen-S and tetric ceram HB. Prepared, unrestored teeth were the weakest group. There was a significant difference between the fracture resistance of intact teeth and the prepared, unrestored teeth. There was also a significant difference among the tested restorative materials. Teeth restored with Admira showed no significant difference when compared with the unprepared teeth. It was concluded that the teeth restored with Admira exhibited the highest fracture resistance.

## INTRODUCTION

Removal of tooth structure *via* cavity preparation has been shown to weaken teeth and increase their susceptibility to fracture[1],[2]. Studies on the weakening of teeth by mesio-occlusal-distal (MOD) cavity preparations and the effect of restorations in strengthening the remnant tissue have been conducted experimentally[3]-[5]. Furthermore, even if fracture does not occur, deflection of a weakened cusp may open the tooth-restoration interface and lead to microleakage resulting in recurrent caries[6]. Depending on the extent of the cavity, restorative treatment is a predisposing factor for an incomplete or complete tooth fracture[7],[8].

Stress concentrates at the internal line angles of the prepared cavity when restorations are not bonded to the tooth and at the dentine-enamel junction for bonded restorations. Therefore, fatigue failure could occur as a result of the masticatory process if the level of stress in these areas was sufficient to initiate crack propagation[9]. Cavity preparation and endodontic treatment can cause higher stress concentration in dentin, compared with vital teeth, but proper restoration can minimize internal stresses[10].

Stabilization (strengthening) of the tooth after an intracoronal preparation can be achieved by covering the outer surface with a cast metal onlay (external splinting). However, this procedure involves additional loss of healthy dental hard tissue[11]. An alternative method to external splinting is the adhesive technique, i.e., “internal splinting or restoration”, which can be used for the stabilization of weakened teeth[12]. Resin-based materials are rapidly becoming the primary restorative material to replace tooth structure lost through dental caries or trauma, although the use of direct composites in restoration of posterior teeth has been problematic[13]. The main problems incurred with posterior composite restorations have been their tendency to form marginal gaps due to polymerization shrinkage and lack of strength. Composites are stressed severely when used for class II filling[14],[15]. Polymerization shrinkage stresses of dental composites are often associated with marginal and interfacial failures of bonded restoration. The magnitude of stress depends on composite composition (filler content and matrix composition) and its ability to flow before gelation[16].

A new generation of composite resins has been introduced to the dental market with new filler designs that permit a more forceful placement into cavity preparations. They are classified by manufacturers as packable posterior composite resins. The difference in the plasticity of the packable composites may make contact adaptation to the dentin bonding agent and walls of the cavity preparations more difficult[17]-[19]. In recent years, new resin composites with reduced polymerization shrinkage and shrinkage stresses have been introduced, i.e., low-shrinkage composite resin and ormocer-based composites. The reduction in polymerization shrinkage decreases problems with contraction stresses, sensitivity, microleakage, recurrent caries and negative pupal sequelae[20]. Ormocer technology relies on an alkoxysilane network, which is chemically attached to traditional methacrylate groups. Initial reports confirmed that ormocer based materials exhibit less shrinkage and have better biological properties than conventional Bis-GMA materials[21],[22].

A significant reduction in the stress levels at the tooth-restoration interface was achieved using optimized cavity design or restoration shapes. This method can provide an efficient means of reducing the stresses in restored teeth, and hence has the potential of prolonging their service lives[6]. The hypothesis of the study was that a new restorative composite resin could improve the fracture resistance of maxillary premolars to a degree comparable with that of intact teeth. The aim of this study was to compare the fracture resistance of premolar teeth restored with different composite filling materials to the fracture resistance of intact teeth and teeth prepared without restoration.

## MATERIALS AND METHODS

### Materials

A total of 50 sound maxillary premolars, extracted for orthodontic purposes, were selected. Ten intact premolars served as the control group and 40 premolars received MOD cavity preparation and were divided into four groups (*n* = 10). Any calculus deposits and soft tissue were removed from the selected teeth using a hand scaler. The teeth were cleaned with pumice and examined under ×10 magnification to detect any preexisting defects. Following post-extraction storage in 10% neutral buffered formalin for at least four days, the teeth were stored in tap water at room temperature until used. The widest buccolingual width (BLW), mesio-distal width (MDW) as well as occluso-gingival width (OGW) dimensions of each tooth were measured and recorded. Selection of teeth was carried out using the average crown dimensions proposed by Galan[23], which include 9.0-9.6 mm BLW, 7.0-7.4 mm MDW, and 7.7-8.8 mm OGW. Each tooth was fixed, with the crown uppermost and long axis vertical, in 1/2 inch polyvinyl chloride (PVC) rings using auto-cured acrylic resin. The level of the resin was limited to 1.0 mm below the cementumenamel junction.

The materials used in this study are presented in ***Table 1***. The total content of inorganic fillers in the Tetric ceram HB composite was 81%(wt) or 63%(vol) and the particle size ranged from 0.04 to 3.0 µm. The total content of inorganic fillers in the InTen-S composite was 74%(wt) or 51%(vol). The particle size ranged between 0.2 and 7.0 µm. The filler content of the Admira was 77% by weight.

**Table 1:** Materials used in this study

Materials	Composition	Lot number	Manufacturers
Excite	Contains HEMA, dimethacrylates, phosphonic acid acrylate, highly dispersed silicon dioxide, initiators and stabilizers in an alcohol solution	F29207	Ivoclar, Vivadent Schaan / Liechtenstein
Tetricceram HB	Bis-GMA, urethane dimethacrylate, decandiol dimethacrylate (19%wt), Barium glass, Ba-Al-fluorosilicate glass, ytterbium trifluoride, highly dispersed silicon dioxide and speroid mixed oxide, catalysts, stabilizers and pigments (0.8%wt).	HO1690	Ivoclar, Vivadent Schaan / Liechtenstein
InTen-S	BisGMA, urethane dimethacrylate, BisEMA [17.5%(wt)], barium glass, ytterbium trifluoride and copolymers, additives, catalysts, stabilizers and pigments [0.6%(wt)]	J21017	Ivoclar, Vivadent Schaan / Liechtenstein
Admira Bond	Acetone, bonding ormocer, dimethacrylate, functionizing, methacrylates, initiators, stabilizer	481012	Voco 27457,
Admira	Ormocer-based resin, Dimethacrylates (Bis-GMA, UDMA, TEGDMA), Campherquinone, Amines, BHT, Inorganic glass fillers	461266	Cuxhaven, Germany Voco 27457, Cuxhaven, Germany

### Methods

**Fig. 1:**
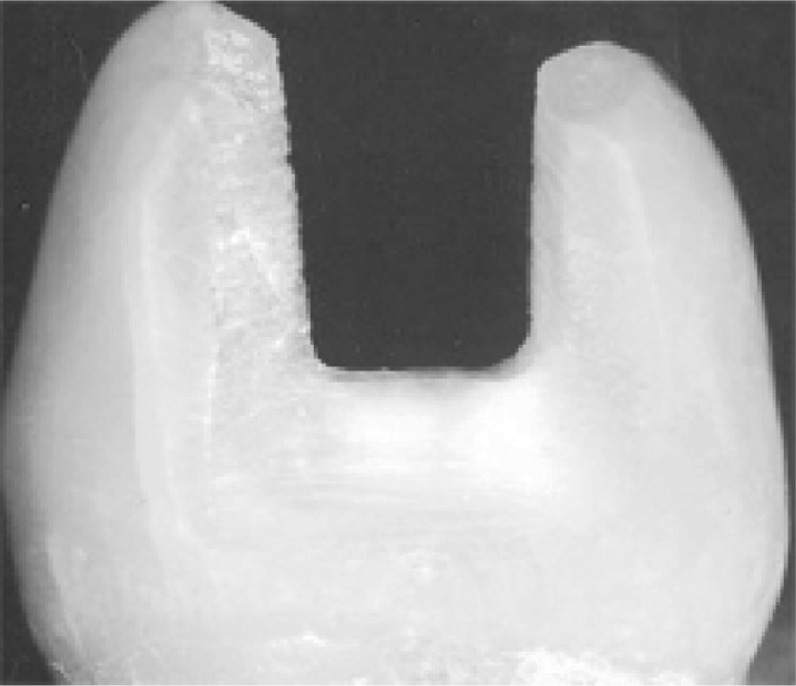
Class II MOD cavity, prepared and unrestored premolar

The teeth were divided randomly into five groups with 10 teeth in each. Group 1: Control, intact, unprepared, and unrestored teeth. Group 2: MOD cavities were prepared and unrestored (***Fig. 1***). Group 3: MOD cavities were prepared and restored with packable resin–based composite (Excite/Tetric–ceram HB). Group 4: MOD cavities were prepared and restored with low-shrinkage resin-based composite (Excite/InTen–S). Group 5: MOD cavities were prepared and restored with Ormoce-based filling material (Admira bond/Admira).

Group 2 MOD cavities were prepared using a tungsten carbide straight fissure bur (FG 172, KERR Haw, Canada) in high-speed water-cooled hand piece. The dimensions of the cavity preparations were such that remaining tooth structure was weakened (***Fig. 1***). The isthmus width of the preparation is one third of the inter-cuspal distance. The width of the proximal box is one third of the total facio-lingual distance. The facial and lingual walls of the occlusal segment were prepared parallel to each other with the cavosurface angle at 90°[24]. The occlusal portion was prepared to a depth of 2 mm. Standardized depth was verified with a scaled periodontal probe (instrument number 23/ UNC 15; Hu Friedy, Chicago, IL, USA). The axial wall in the proximal box was prepared to a depth of 1.5 mm and the gingival margin was placed 1 mm occlusal to the cementum-enamel junction. The preparations were finished to exact dimensions using a parallel-sided round ended bur (DK Holdings, Staplehurst, UK), without water coolant, in a laboratory hand piece at a maximum speed of 8000 *g*. The internal line and point angles were rounded[25]. The measurements were checked using RS Fernier calipers.

The prepared cavities in Group 3 were dried with oil-free air and etched for 30 sec using 37% phosphoric acid gel (Total Etch), rinsed with water spray for 30 sec and gently air dried. The prepared cavity surfaces were saturated with a generous amount of Excite adhesive resin using a Iovclar Vivadent applicator gently agitated onto all the prepared dentin surfaces for 10 sec and light-cured for 20 s with halogen light curing unit (Astralis 7, low power program 400 mw/cm^2^, Martin Medizin Technik Cebruder Martin GmbH, Germany). An Atofflemire (FG,9714, Kerr Hawe, Canada) retainer system was used with ultra thin (0.001 inch) universal metal matrix bands that were changed for each restoration. Tetric ceram HB composite resin was placed in 2.0 mm increments and polymerized for a 40 sec increment with a final cure of 40 sec.

The prepared cavities in Group 4 were etched and bonded as group 3. After application of the matrix system, InTen-S resin-based composite was applied in 2.0 mm thick layers and adapted with a suitable plastic instrument. Each layer was cured for 20 s using the Astralis 7 high performance light curing program (750 mw/cm^2^) according to the manufacturers' instructions.

The prepared cavities in Group 5 were etched as in Group 3 and Admira bond was applied onto the prepared surfaces using a disposable brush. After 30 sec, it was dispersed with faint air-jet and light cured for 20 sec using light curing unite (Astralis 7,400 mw/cm^2^). After application of the Toffelmire retainer system with ultra thin metal band, Admira was applied in increments of 2.5 mm thickness until the cavity was filled. Each increment was cured for 40 sec. The matrix band was removed and a final cure of 40 sec was done.

**Fig. 2:**
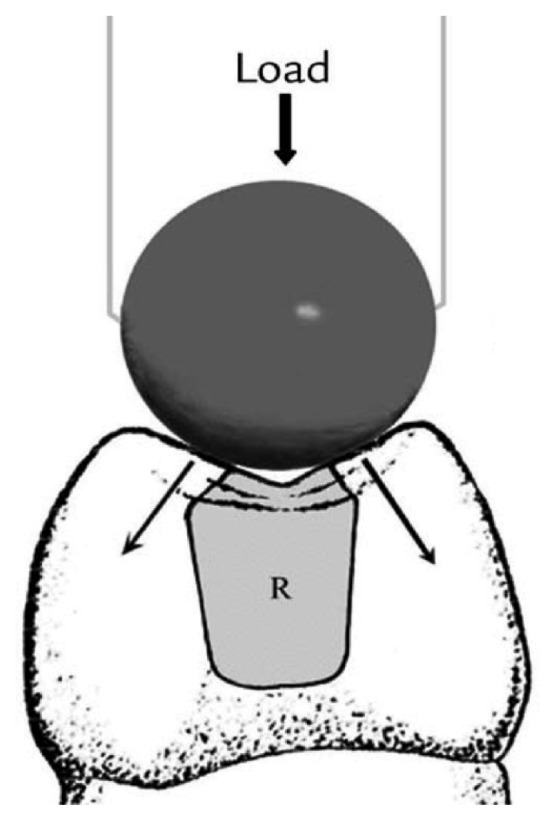
Schematic diagram of the applied load to the tested specimen

All the restored specimens were finished using a long-tapered-trimming, fine-finishing bur (FG,9714, Kerr Hawe, Canada).The specimens were stored in distilled water and thermo cycled for 5,000 cycles at 5°C and 55°C with each cycle corresponding to a 15 sec bath at each temperature[26]. The specimens were tested individually in a universal testing machine (Lloyd Instruments LTD, Farcham Hants UK). Each specimen was subjected to compressive loading using a 4.8 mm diameter steel ball at a crosshead of 2 mm/min. The ball should contact the inclined planes of the facial and palatal cups beyond the margins of the restorations (***Fig. 2***). Peak load to fracture (kg·f) was recorded for each specimen and the mean was calculated for each group.

### Statistical analysis

Data were analyzed using one-way analysis of variance (ANOVA) test to access the difference between numerical groups. LSD test was used as a pair-wise test to examine the difference between the continuous numerical values. All statistical analyses were performed using SPSS 10 for Windows (SPSS Inc IL, USA) and Microsoft Excel, Office XP software (Microsoft Corporation IL, USA), a *P* < 0.05 was considered as difference.

## RESULTS

The mean fracture load (kg · f) for all groups is presented in ***Table 2***. ANOVA revealed that there was a significant difference between all groups at *P* < 0.001 (***Table 3***). LSD test showed that there were significant differences (*P* < 0.05) between the unprepared and all prepared teeth, either restored or unrestored, except those restored with Admira at *P* > 0.05 (***Table 2***). Additionally, there were significant differences in fracture resistance between the prepared, unrestored teeth, and those restored with Tetric Ceram HB, InTen-S resin-based composite or Admira restorative material (*P* < 0.05). There was a significant difference between the prepared teeth restored with Tetric-Ceram HB and the other groups, either restored or unrestored (*P* < 0.05).

**Table 2:** Mean fracture load (kg·f) of restored and unrestored teeth

Group	Mean fracture load (kg • f)	
1. Control (intact, unprepared and unrestored teeth)	177.22±14.94	11.56
2. Prepared and unrestored teeth	53.21±10.48*	
3. Prepared and restored teeth with Tetric-Ceram HB	96.85± 6.57*	
4. Prepared and restored teeth with InTen-S	127.32±15.93*	
5. Prepared and restored teeth with Admira	175.31±13.89	

*P < 0.05.

(mean±SD)

**Table 3:** One-way analysis of variance (ANOVA) test

	Degree of freedom	Sum of squares	Sum of squares	*F*	*P*
Between	4	112,206.02	28,051.50	170.443	0.000*
Within	45	7406.09	164.58		
Total	49	119,612.10			

*P< 0.001.

## DISCUSSION

A fracture is a complete or incomplete break in a material resulting from the application of excessive force. Fracture resistance is an important property directly related to cracking. Experimental and theoretical efforts have been made to relate the strength of a material to its fracture resistance in addition to the structural parameters[27]. Strictly speaking, resistance to fracture in a composite would depend on the specific failure mode of heterogeneous materials[28].

Depending on the stress state in a composite material, both an increase and decrease of fracture resistance can be observed as the strength varies. There is no satisfactory model that can explain the variety of dependence of fracture resistance on the strength of a composite and the cause of its complex inhomogeneous character. A relation is established for the dependence of fracture resistance on both the critical deformation energy density and minimum size of structural element at the onset of global failure of a micro inhomogeneous material[29]. Masticatory forces on restored or unrestored teeth have a tendency to deflect the cusps under stress[3]. Even though *in vitro* studies are not an actual reproduction of a typical chewing stroke, in that they apply a continuously increasing force until the tooth fractures, they represent an important source of information on the structural integrity of the tooth. They also identify the weakest component, whether it is inherent properties of the restoration or the fatigue of the brittle tooth tissues at the adhesive interface. Clinically, masticatory forces are of a relatively consistent magnitude and applied over a longer period of time. They vary in speed of application and direction[30] and contribute to a different pattern of fracture when it occurs[31]. Cavity preparations with rounded internal line angles result in reduced stress concentration in the dentin at the angles, avoiding the occurrence of fatigue when there is an effective increase in the cusp height and decrease in the cusp width[25].

Since the introduction of composite resin restorative materials in the 1960, these widely used materials have been the subject of numerous studies to improve their properties. Composite resin restorations retained with an adhesive resin are the most popular restorations currently used[32]. Composite resins have mechanical properties similar to dentin[33]. Much attention has been focused on the polymerization shrinkage of these materials. If the polymerization shrinkage is great enough, the resulting stresses can compromise the union (chemical bonding and/or micromechanical interlocking) of the composite with the cavity surfaces of the tooth. If the polymerization stresses exceed the strength of the composite-tooth bond, bond breaking occurs and causes a gap to form between the tooth and the restoration[34]. If the amount of polymerizing material in composite restorations could be reduced, the detrimental problem of polymerization shrinkage would be decreased[35].

Denehy and Torney[36] were the first authors who proposed the use of adhesive materials to reinforce weakened teeth and support undermined enamel. Bremer and Geurtsen[37] have shown that the weakening effect of preparation can be alleviated with the use of adhesive materials. These materials not only seal the margin but also increase the retention and resistance properties of the restored tooth[37]. Composite restorations that are bonded to tooth structure have been shown to reduce deformation of cusps under occlusal loading[38]. Composite resin restorations showed the most favorable stress distribution pattern in MOD cavity restorations in both vital and endodontically treated teeth[10].

The results of this study showed that Admira filling material has the highest fracture resistance when compared to the other restorative materials because it is based on ormocer technology, which should not be confused with glass ceramic fillers in conventional composites. Ormocers consist of a long “backbone” of silicon instead of carbon, on which carbon-carbon double bond-containing side-chains are grafted. The larger size of the monomer molecule can reduce polymerization shrinkage and wear, and reduce leaching of monomers, which makes ormocers a material of interest for use as a matrix for resin composites[21]. Like all composites, Admira also contains conventional filler particles made of glass and ceramic. These three-dimensional polymeric composites form an innovative resin fraction and replace a large part of the organic resin matrix of conventional composite, thereby decreasing polymerization shrinkage (1.97% by volume, according to manufacturer data)[39],[40].

The findings of this study show that the stabilization of a prepared tooth can be achieved with a restoration adhesively bonded to prepared cavity walls. However, not all materials included in this experiment resulted in fracture resistance similar to healthy teeth. It has been reported that restorations that merely fill the preparation without adhesion, such as amalgams or gold inlays, do not reinforce weakened tooth structure[41]-[43]. A significant reduction in the stress levels at the tooth-restoration interface, where bonding is imperfect was achieved using optimized cavity design or restoration shapes[6].

This study showed that teeth restoration with Ormocer-based filling material also strengthened the remaining tooth structure. No significant difference in the fracture resistance was found between teeth restored with Ormocer-based filling material and the unprepared teeth. This result could be attributed to low polymerization shrinkage of Ormocer compared to conventional composite resin and the decreased polymerization stresses. Burgoyne *et al*.[44] reported that, despite the improved characteristics of posterior composite resins, they still show relatively high polymerization shrinkage of 2.6%-7.1% by volume.

The results of this work indicate that teeth restored with packable composite resin exhibited inferior numerical values of fracture resistance in relation to the groups restored with low-shrinkage composite, Ormocer-based filling material and unprepared teeth. Low-shrinkage composite exhibits one of the lowest polymerization shrinkage and shrinkage stress values, providing less deformation of tooth structure during the composite polymerization and improving marginal quality[43],[45].

In these trials, it was found that the cavity preparation weakens the teeth. On the basis of the application of static occlusal loading, the Ormocer-based restoration (Admira) increased the resistance to fracture similarly to that of the intact, unprepared teeth. Moreover, restoring teeth with low-shrinkage resin-based composite (InTen-S) increased the fracture strength up to 72% of the mean value exhibited by the intact teeth. Restoring teeth with packable resin-based composite (Tetric-ceram HB) increased the fracture strength up to 54.5% of the mean value exhibited by the intact group.

Very contradictory findings have been published about the effect of adhesive resin-based composite restorations on fracture resistance. Whereas George *et al*.[46] ascribed a significantly lower fracture resistance to teeth which were adhesively restored with resin-based composite with or without dentin bonding agent. No findings have been published about the effect of Ormocer-based composite and low-shrinkage composite restorations on fracture strength[46].

In conclusion, within the limitation of this study, under compression loading, the use of Admira and InTen-S restorative materials significantly strengthen maxillary premolars with MOD preparation.

## References

[1] Joynt RB, Davis EL (1989). Fracture resistance of posterior teeth restored with galss–ionomer–composite resin system. J Prosthet Dent.

[2] Stephan EW, Staninec M, Lacy AM (1992). Effect of bonded amalgam on the fracture resistance of teeth. J Prosthet Dent.

[3] Jagadish S, Yogesh BG (1990). Fracture resistance with class II silver amalgam, posterior composite and glass–ionomer cement restorations. Oper Dent.

[4] Burk FJT, Wilson NI, Watts DC (1993). The effect of cavity wall taper on fracture resistance of teeth restored with resin composite inlays. Oper Dent.

[5] Brunton PA, Paul AB, Paul C, Trevor B, Nairn HFW (1999). Fracture resistance of teeth restored with onlays of three contemporary tooth–colored resin-bonded restorative materials. J Prosthet Dent.

[6] Couegnat G, Fok SL, Cooper JE, Alison JE, Qualtrough AJ (2006). Structural optimization of dental restorations using the principle of adaptive growth. Dent Mater.

[7] Sheth JJ, Fuller JL, Jensen ME (1988). Cuspal deformation and fracture resistance of teeth with dentin adhesives and composites. J Prosthet Dent.

[8] Jokstad A, Mjör IA, Qvist V (1994). The age of restoration in situ. Acta Odontol Scand.

[9] Thompson S (2002). Investigation into the magnitude and placement of the stress at the tooth–restoration interface using finite element analysis.

[10] Jiang W, Bo H, Yongchun G, LongXing N (2010). Stress distribution in molars restored with inlays or onlays with or without endodontic treatment: A threedimensional finite element analysis. J Prosthet Dent.

[11] Bouillaguet S, Watalta JC (2004). Future Directions in bonding resins to the dentine-pulp complex. J Oral Rehabilit.

[12] Joynt RB, Wieczkowski G, Klockowski R, Davis EL (1987). Effect of composite restorations on resistance to cuspal fracture in posterior teeth. J Prosthet Dent.

[13] Paulo HP (2002). Fracture of teeth directly and indirectly restored with composite resin and indirectly restored with ceramic materials. Am J Dent.

[14] Tay FR, Gwinnett AJ, Pang KM, Wei SH (1995). Variability in micro leakage observed in total each wet bonding technique under different handling conations. J Dent Res.

[15] Bown RI, Nemoto K, Rapson OE (1983). Adhesives of various materials to hard tissues: Force developing in composite materials during handling. JADA.

[16] Braga RR, Ferracan DL (2004). Alternatives in polymerization contraction stress management. Crit Rer Oral Bio Med.

[17] Meieres Je, Kaxmi G, Rand Meier D (2001). Microleakage of packable composite resins. Oper Dent.

[18] Loguercio AD, de Oliveira Bauer JR, Reis A, Grande RH (2004). In vitro microleakage of packable composition in class II restoratons. Quint Int.

[19] Hagashi M, Wilson NH, Watts DC (2003). Quality of marginal adaptation evaluation of posterior composite in clinical trials. J Dent Res.

[20] Canon ML (2003). Advances in pediatric esthetic dentistry. Compend Contain Educ Dent.

[21] Bouillaguet S, Shaw L, Gonzalez L, Wataha JC, Krejci I (2002). Long term cytotoxicity of resin- based dental restorative materials. J Oral Rehabil.

[22] Franz A, Franz K, Margit A, Xiaohui RF, Gabriele G, Wolf DR (2003). Cytotoxic effect of pack able and non packable dental composites. Dent Mater.

[23] Galon Jr (1970). Contribution to the study of the main dimensions of human permanent teeth from white Brazilians of both genders. Rev Bras Odont.

[24] St-Georges AJ, Sturdevant JR, Swift EJ, Thompson Y (2003). Fracture resistance of prepared teeth restored with bonded inlay restorations. J Prosthet Dent.

[25] Johnson EW, Castaldi Cr, Gau DJ, Wvsocki GP (1968). Stress pattern variations on operatively prepared human teeth studied by three- dimensional photo-elasticity. J Dent Res.

[26] (1994). International Organization for standardization ISO TR 11450.

[27] Schultrich B (1986). Strength of cemented carbides, in: Mechanical properties of brittle materials, in: Modern theories and experimental evidence (Wiss. Acad. Tier Wissen-schaften der DDR. Zentralinstitut fur Festkorperphysik und Werkstofforschung N32.

[28] Sih GC (1991). Mechanics of Fracture Initiation and Propagation.

[29] Lugovy MI, Podrezov YuN, Slyunyaev VN, Verbylo DG (1999). Fracture resistance and strength of two-phase WC–Ni alloy. Theor Appl Fract Mec.

[30] Eakle WS (1986). Incereased fracture resistance of teeth: Comparison of five bonded composite resin systems. Quint Int.

[31] Cavel WT, Kelsey WP, Blankenau RG (1985). An *in vitro* study of cuspal fracture. J Prosthet Dent.

[32] Sadowsky SJ (2006). An overview of treatment considerations for esthetic restorations:a review of the literature. J Prosthet Dent.

[33] Willems G, Lambrechts P, Braem M, Celis JP, Vanherle G (1992). A classification of dental composites according to their morphological and mechanical characteristics. Dent Mater.

[34] Marin D, Delong R, Douglag WH (1984). Cusp reinforcement by the acid- technique. J Dent Res.

[35] Reeh ES, Douglas WLL, Messer HH (1989). Stiffness of endodontically–treated teeth related to restoration technique. J Dent Res.

[36] Denehy GE, Torney DL (1976). Internal enamel reinforcement through micromechanical bonding. J Prosthet Dent.

[37] Bremer BD, Geurtsen W (2001). Molar fracture resistance after adhesive restoration with ceramic inlays or resin-based composites. Am J Dent.

[38] Boyer DB, Roth L (1994). Fracture resistance of teeth with bonded amalgams. Am J Dent.

[39] Wolter H, Storch W, Ott H (1994). New inorganic / organic co-polymers( ORMOCERS) for dental applications. Mater Res Soc Symp Proc.

[40] Watts DC (1998). Preliminary measurements of polymerization shrinkage strain in definite™ ormocer restoratives, in degussa dental forschung. Interner Forschungsberichi.

[41] Studer SP, Wettstein F, Lehner C, Zullo TG, Scharer P (2000). Long-term survival estimates of cast gold inlays and onlays with their analysis of failures. J Oral Rehabil.

[42] Eakle WS, Staninec M (1992). Effect of bonded gold inlays on fracture resistance of teeth. Quint Int.

[43] Sntini A, Milpia E (2004). Microleakage around a low- shrinkage composite cured with high performance light. Am J Dent.

[44] Burgoyne AR, Nicholls JI, Brudvik JS (1991). In vitro two-body wear of inlay-only composite resin restorations. J Prosthet Dent.

[45] Civelek A, Erosy M, L'Hotelier E, Sovman M, Say EC (2003). Polymerization shrinkage in calss II cavities of various resin composite. Oper Dent.

[46] Ceurtsen W, Garcia-Codoy F (1999). Prevention and treatment of the cracked tooth syndrome by bonded restorations. Am J Dent.

